# Automated Analysis of Stroke Mouse Trajectory Data With Traja

**DOI:** 10.3389/fnins.2020.00518

**Published:** 2020-05-25

**Authors:** Justin Shenk, Klara J. Lohkamp, Maximilian Wiesmann, Amanda J. Kiliaan

**Affiliations:** Department of Anatomy, Radboud University Medical Center, Preclinical Imaging Centre PRIME, Radboud Alzheimer Center, Donders Institute for Brain, Cognition, and Behavior, Nijmegen, Netherlands

**Keywords:** animal tracking, neuropsychiatric disorders, machine learning, home-cage, mouse, stroke

## Abstract

Quantitative characterization of mouse activity, locomotion and walking patterns requires the monitoring of position and activity over long periods of time. Manual behavioral phenotyping, however, is time and skill-intensive, vulnerable to researcher bias and often stressful for the animals. We present examples for using a platform-independent open source trajectory analysis software, Traja, for semi-automated analysis of high throughput mouse home-cage data for neurobehavioral research. Our software quantifies numerous parameters of movement including traveled distance, velocity, turnings, and laterality which are demonstrated for application to neurobehavioral analysis. In this study, the open source software for trajectory analysis Traja is applied to movement and walking pattern observations of transient stroke induced female C57BL/6 mice (30 min middle cerebral artery occlusion) on an acute multinutrient diet intervention (Fortasyn). After stroke induction mice were single housed in Digital Ventilated Cages [DVC, GM500, Tecniplast S.p.A., Buguggiate (VA), Italy] and activity was recorded 24/7, every 250 ms using a DVC board. Significant changes in activity, velocity, and distance walked are computed with Traja. Traja identified increased walked distance and velocity in Control and Fortasyn animals over time. No diet effect was found in preference of turning direction (laterality) and distance traveled. As open source software for trajectory analysis, Traja supports independent development and validation of numerical methods and provides a useful tool for computational analysis of 24/7 mouse locomotion in home-cage environment for application in behavioral research or movement disorders.

## Introduction

Rodent locomotion has been studied in the context of various disease models such as spinal cord injury (Barrière et al., 2008; Tester et al., 2011, 2012), neurodegenerative diseases such as Parkinson’s (Morris et al., 1996, 2001; Amende et al., 2005) and Down syndrome (Hampton et al., 2004; Herault et al., 2017), assessment of pharmacological agents (Masocha and Parvathy, 2009), genetic mutations (Bothe et al., 2004; Crone et al., 2009), and stroke (Encarnacion et al., 2011; Hetze et al., 2012). Locomotion monitoring has been used both as a proxy for measuring illness and fatigue as well as overall development and recovery. Automated quantitative analysis of mouse phenotype allows researchers to objectively assess cognitive and motor abilities and disturbances brought about by genetics, disease processes, and interventions. The ability of these effects to be observed, measured and communicated, is constrained by the availability of assays which reflect physiological and cognitive changes occurring over indeterminate time intervals. Further, the time resolution of observations and analysis are limited by the availability of behavioral data and analytical methods. Sharing data and analytical methods allows for increasing the validity of experimental modalities.

Phenotyping mouse models typically involves screening mice through various behavioral tests to measure anxiety, learning, memory, or locomotion in experimental setups outside the home-cages. Several mouse tracking tools exist for phenotyping; a recent review can be found at Chaumont et al. (2018). These tools, however, either are not designed to be used in home-cage environments or require expensive commercial software which limits the reproducibility of data collection and analysis. Monitoring in artificial environments introduces an additional stress and discomfort to the animals, and the short-term nature of the experiments risk missing important behavioral patterns which can only be discovered during long-term observation.

The Open Field (OF) test is a widely used experimental paradigm for quantifying mouse locomotion and monitoring behavior. It allows providing descriptive statistics of mobility and stress-related behaviors such as tendency to remain near walls or corners (Benjamini et al., 2010). OF is limited, however, because it is subject to experimenter bias, requires trained animal handler’s to be present, management of equipment, and specialized space for experimentation. Multiple exposure to the OF environment leads to habituation, which decreases exploratory behavior and can mask recovery after an ischemic insult. In addition, the novelty of the environment causes stress to the animal which may affect the observed response. Similarly, the Corner Test (Balkaya et al., 2013) is widely used to identify sensory-motor functional deficits like laterality preference after an experimental stroke, but suffers the same drawbacks.

Reproducibility of movement data analysis depends on widespread access to the data and analytical methods for verification within the scientific community (Benjamini et al., 2010). The vast majority of researchers use commercial software for recording and digitally analyzing the OF test results. A number of applications capture locomotion with video (Casadesus et al., 2001; Bailoo et al., 2010) or photo-beams (Dunne et al., 2007). Most tracking systems require specialized cages, illumination (Spink et al., 2001; Reeves et al., 2016) or the presence of intrusive devices which do not fit within standard mouse home-cage and interfere with mouse behavior, or are based upon proprietary methods or software specific to the data sources. Such experimental setups rely on a novel environment during recording, or are otherwise difficult to scale to large studies without considerable investment in equipment.

Ability to customize parameters used for analysis is a priority in defining quantitative statistics, since the validity of the construct in relation to the features of interest in many cases cannot be determined empirically (Noldus et al., 2001). For example, laterality is defined in various ways in the literature, e.g., with various thresholds for distance traveled or angular movement. Software which allows customizing the parameters for analysis supports independent verification of the results and fine-tuning of analytical methods for increased internal validity (Noldus et al., 2001).

Open source software has been developed in the past years for tracking mice in OF (M-Track; Reeves et al., 2016) and other specialized cage environments (Live Mouse Tracker; Chaumont et al., 2018). Singh et al. (2019) developed a camera-based system for mouse tracking for mouse home-cage locomotion tracking and analysis. Their setup requires the user to be experienced in configuring hardware and does not provide tools for analyzing the distance traveled.

Trajr is an R package developed for analysis of animal movement in two-dimensional space (Mclean and Volponi, 2018). It provides several methods for trajectory analysis and data preprocessing. The source code, however, is not optimized for analysis of the millions of data points needed to track the lifetime position of mice, the most common used experimental animal model. Further, there are many advanced data modeling tools for machine learning, such as TensorFlow and PyTorch, which are not accessible in R, thus unnecessarily restricting the user to traditional analytical techniques.

The Python programming language, on the other hand, is a general-purpose programming language and is the primary language used for implementation of current state-of-the-art trajectory prediction models [e.g., Social Ways (Amirian et al., 2019), Next (Liang et al., 2019), and TraPHic (Chandra et al., 2018)]. A library which bridges the most widely used machine learning packages with mouse home-cage trajectory is thus needed.

We present a Python package, Traja, for automated analysis of activity and position extracted via the 12 capacitive home-cage sensors in the DVC [Tecniplast S.p.A., Buguggiate (VA), Italy] (Iannello, 2019; Pernold et al., 2019) to quantify several behavioral modalities in a stroke mouse model. Stroke is a motor impairment disease; therefore, Traja is a suitable application for identifying changes in behavior and motor function relevant to surgical intervention and treatments like diet and exercise (Calahorra et al., 2019). We analyze mouse activity, distance, velocity, and turning bias/laterality from data collected in a comparison of a group on Control diet and a group treated with the multinutrient intervention, Fortasyn (Wiesmann et al., 2018). Fortasyn consists of fatty acids, phospholipids, and vitamins stimulating neuronal membrane formation, and improving vascular health by increasing cerebral blood flow (CBF) which suggest that this diet may also improve damages caused by cerebrovascular diseases (CVD) such as stroke. This study on the impact of Fortasyn on behavior (Open Field, Pole test), neuroimaging and post-mortem brain measures was previously published (Wiesmann et al., 2018). In short, the study provides evidence that Fortasyn leads to improved brain integrity, sensorimotor integration and neurogenesis, while motor skills did not recover in female stroke mice on Fortasyn diet (Wiesmann et al., 2018). The present results computed by Traja are a valuable addition to the previously performed study, since DVC derived data might pick up more effects than classical behavioral tests (e.g., Open Field). We demonstrate the capability of metrics derived from home-cage activity and position tracking to study differences in the neurobehavioral phenotypes of mice over extensive lengths of time, within a method that is accessible to researchers possessing moderate programming background. Thus, by making use of the DVC dataset of this female stroke mouse model, we show that the Traja software package is a valid method for semi-automated trajectory analysis.

## Materials and Methods

### Description of the Traja Python Package

The user of the Python package Traja can carry out data selection by using filter settings like minimal distance moved to eliminate slight “apparent” movements. Several quantitative measures of behavior are available and can be described with descriptive statistics relevant to neurobehavioral research, including activity, distance, velocity, turn angle, and laterality. A list of functionalities and specifications of Traja are listed in Supplementary Tables 1, 2.

### Automated Locomotion and Trajectory Analysis

#### Activity and Centroids

Activity and centroids were measured by sensing boards, equipped with 12 capacitive-based electrodes, underneath each Digital Ventilated Cage [Tecniplast S.p.A., Buguggiate (VA), Italy] as previously described in detail (Iannello, 2019; Pernold et al., 2019). In short, proximity sensors are able to measure electrical capacitance of each electrode in 250 ms time intervals 24/7. As soon as an animal is moving in the cage, the electrical capacitance of the proximity sensors are influenced by the dielectric properties of matter in close proximity to the electrode. Consequently, animals moving across the electrodes are detected and recorded as change in capacity over a limited time interval. An activity event describes the absolute value of the difference between two consecutive measurements for each electrode that is compared with a set threshold to control for noise. Centroids include the x, y coordinates of the mouse position inside the cage. For the x, y-value calculation, the mouse position is estimated in the average position between the centers of the active electrodes weighted by using change in capacity, as described elsewhere in more detail (Iannello, 2019).

#### Distance and Velocity

Distance and velocity were calculated using the first and second derivative, respectively, of the centroid coordinates with respect to time (Iannello, 2019). The distance computed with Traja was matching the distance obtained with Ethovision XT 14, indicating that Traja accurately computes trajectory parameters (Supplementary Figure S1). In Traja this is accomplished with traja.calc_displacement() and traja.calc_derivatives(), respectively. Velocity was measured with a minimum velocity threshold of 0.02 m/s.

#### Turn Angle and Laterality

Angular velocity has been used in several animal models including mice and fish (Williams et al., 2017) for observing reflexes and locomotion. Extending the nomenclature in Noldus et al. (2001) and Spink et al. (2001) with heading at time step *n* as *HE*_n_ and time coordinate, *R* relative turn angle *RTA*_n_ = *HE*_n_−*HE*_n−1_ and relative angular velocity $R\,A\,V_{\text{n}}=\frac{R\,T\,A_{\text{n}}}{t_{\text{n}}-t_{n-1}}$,we calculate laterality index $L\,I=\frac{R}{R+L}$ where *R* is the number of right turn angles *RTA* ∈ [30, 90], *L* is the number of left turn angles *RTA* ∈ [−90, −30], and *LI* ∈ [0, 1] and the minimum velocity is 1 cm/s. Turn bias of trajectories can be visualized using traja.polar_bar().

### Stroke Disease Model

All results regarding behavioral tests, neuroimaging, and post-mortem brain analysis of the dietary intervention Fortasyn in a female stroke mouse model were published earlier by Wiesmann et al. (2018). The focus of the present study is the trajectory analysis of the female stroke model in DVC with the software Traja.

#### Transient Middle Cerebral Artery Occlusion

Ischemic stroke was induced in female C57BL/6JRj mice by a transient middle cerebral artery occlusion (tMCAo), which is mimicking one of the most common types of ischemic stroke in patients (Engel et al., 2011). The intraluminal occlusion model was performed as described elsewhere with minor modifications (Engel et al., 2011; Zinnhardt et al., 2015). In short, a 7–0 monofilament (tip diameter 190–200 ml, coating length 2–3 mm, 70SPRePK5, Doccol Corp., Sharon, MA, United States) was inserted in the right common carotid artery and placed to block blood supply via the middle cerebral artery. The filament was held in place for 30 min followed by retraction of the filament leading to reperfusion. A Laser Doppler probe (moorVMS-LDF2, Moor Instruments, United Kingdom) was placed on the skull of the mice to monitor cerebral blood flow, considering a drop of ≥ 80% CBF as a successful stroke induction. Animals were anesthetized during the whole time of surgery, using 1.5% isoflurane (Abbott Animal Health, AbbottPark, IL, United States) in a 2:1 air and oxygen mixture.

#### Animals, Diet, Housing, and Study Design

**FIGURE 1:**
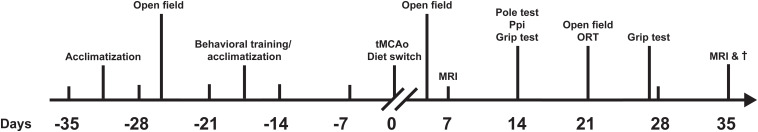
Study design. Acclimatization, behavioral training and baseline behavioral experiments started 35 days before tMCAo. Immediately after stroke induction (day 0), diets were switched to either Fortasyn multinutrient diet (*n* = 11) or to an isocaloric diet (*n* = 12). Additionally, all animals were individually housed in DVC to monitor their locomotive behavior, including activity, distance moved, velocity, turns and laterality. Until 35 days post-surgery, behavioral tests [pole test, prepulse inhibition (PPI), grip test, Open Field, novel object recognition test (ORT)] were performed and animals underwent two MRI scanning sessions.

At 3–4 months of age, 24 female C57BL/6JRj mice (Harlan Laboratories Inc., Horst, the Netherlands) arrived at the preclinical imaging center of the Radboud university medical center (Radboudumc) (Nijmegen, the Netherlands) where all experiments were performed (see Figure 1 for study design). Animals were group housed (four animals per cage) in DVC [Tecniplast S.p.A., Buguggiate (VA), Italy] which contained corn based bedding material (Bio Services, Uden, The Netherlands), wood wool nesting material (Bio Services, Uden, The Netherlands), and a mouse igloo (Plexx, Elst, The Netherlands). Standard food pellets (Ssniff rm/h V1534−000, Bio Services, Uden, The Netherlands) and autoclaved water were available *ad libitum*. The room had constant temperature (21 ± 1°C), humidity (55 ± 10%), background music and an artificial 12 h light-dark cycle (light on at 7 a.m.). After letting the animals acclimatize, baseline behavioral measurements were performed pre-stroke. All parameters measured in the Open Field (walked distance, velocity, manual scored behaviors) and grip strength test did not differ between the Control and Fortasyn group prior surgery (Wiesmann et al., 2018). Immediately after tMCAo, mice were randomly divided into two experimental groups using a random sequence generator switching from normal chow (Ssniff rm/h V1534-000, Bio Services, Uden, The Netherlands) to either a multinutrient intervention Fortasyn diet (*n* = 12) or an isocaloric Control diet (*n* = 12) (Wiesmann et al., 2018). Both the Fortasyn and the Control diet were based on AIN−93M (Reeves et al., 1993) with 5% fat, but differed with respect to their fatty acid composition and some additional nutrients. The Fortasyn diet contained 0.1% coconut oil, 1.9% corn oil, and 3.0% fish oil, while the Control diet contained 1.9% soy oil, 0.9% coconut oil, and 2.2% corn oil. Furthermore, the Fortasyn diet contained a specific multinutrient composition comprising uridine, omega−3 polyunsaturated fatty acids (PUFAs), choline, B vitamins, phospholipids, and antioxidants (the specific composition is specified in Wiesmann et al. (2017). Both diets were manufactured and pelleted by Ssniff (Soest, Germany) and stored at −20°C until use. Group sizes were calculated based on the effect sizes (Type I error: 0.05, statistical power: 0.80), exclusion and mortality rates determined in our previous study (Wiesmann et al., 2017). Before and 1 day after stroke surgery, all mice were injected Carprofen (Rimadyl, Pfizer Animal Health, Cappele aan de IJssel, the Netherlands) subcutaneously adjusted to their weight (0.1 mL Carprofen l per 10 gram) to prevent discomfort. After surgical intervention, the animals were housed separately in clean DVC to optimize healing of surgical wounds. During the post-surgery period (35 days) it was not necessary to clean the cages due to single-housing. Furthermore, physiological parameters as well as stroke related disturbances in motor function during the recovery period were monitored for each individual mouse. Body weight did not differ between experimental groups, however, during the first week post-stroke, Fortasyn fed mice ate significantly more than Control animals although the diets were isocaloric. No further diet effects on both body weight and food intake were found during the poststroke weeks 2–5. Body weight increased in weeks 2 and 3 and stabilized in week 3 and 5 to baseline levels (Wiesmann et al., 2018).

#### Ethics Statement

Our study was in concurrence with the European regulations on ethics and responsible conduct regarding scientific communication as previously described (Wiesmann et al., 2018). Experiments were performed according to Dutch federal regulations for animal protection and the European Union Directive of 22 September 2010 (2010/63/EU). They were approved and pre−registered by the Animal Ethics Committee (called the Dierexperimentencommissie; DEC, RU−DEC 2014−171) of the Radboudumc. Furthermore, our experiments were performed according to the (updated) recommendations made by the Stroke Therapy Academic Industry Roundtable (STAIR) for the preclinical development of therapies for ischemic stroke (Fisher et al., 2009) and ARRIVE guidelines (Kilkenny et al., 2010). All applicable international, national, and institutional guidelines for the care and use of animals were followed. Our study was also in concurrence with the European regulations on ethics and responsible conduct regarding scientific communication.

### Data Analysis

Mouse tracking was performed using DVC collecting data 24/7 every 250 ms via capacitance sensors placed underneath the home-cage. The raw data of mouse position centroids and activity were provided by Tecniplast. The data was analyzed with Traja.

#### Coding

Traja was written using Python 3.6 and several Python libraries for data management and analysis, with links and descriptions provided in Supplementary Table S2.

#### Software

The software and documentation for Traja are freely available for download at: http://traja.readthedocs.io or from the repository https://github.com/justinshenk/traja (Shenk, 2019). It is compatible with Microsoft Windows, Mac OSX and Linux.

#### Statistical Analysis

Before analysis, data was aggregated for Fortasyn and Control groups. Unless otherwise stated, data were pooled within experimental groups by days from surgery and split into nighttime (7 p.m. – 7 a.m., light off) and daytime (7 a.m. – 7 p.m., light on). Furthermore, effects found across 24 h are considered as overall effects. All data are presented as mean ± SEM, unless otherwise stated. Statistical significance was set at *p* < 0.05. Effects and interactions of longitudinal data were calculated by generalized linear models treating time and diet as fixed effects with the Python statsmodel software package (program items are in Supplementary Table S2). Moreover, for our GEE regression additional statistical measures are provided in the Supplementary Material (e.g., number of observations/clusters, min./max./mean cluster size, skew and kurtosis, standard errors, z-values) clarifying our statistical approach.

## Results

### Activity

**FIGURE 2:**
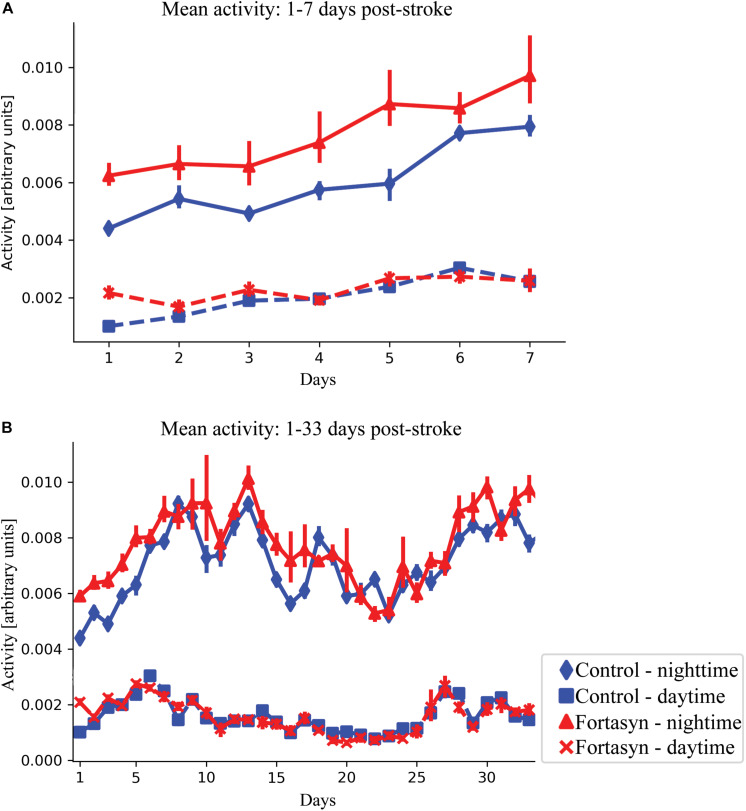
Mean activity measured in Control and Fortasyn fed animals during day- and nighttime between day ranges 1–7 **(A)** and 1–33 **(B)**. **(A)** Fortasyn fed animals were overall significantly more active between day 1–3 (*p* < 0.050). **(A,B)** In both groups, activity increased over time during daytime [days: 1–3 (*p* < 0.001), days: 1–7 (*p* < 0.001)], nighttime [days: 1–7 (*p* < 0.001), days: 1–33 (*p* < 0.017)], and overall (days: 1–7 *p* < 0.001). During daytime activity decreased in Control and Fortasyn animals between day 1–33 (*p* < 0.020).

Recovery following stroke induction was monitored by analyzing mouse activity during day- and nighttime. In the first 3 days after surgery, Fortasyn fed animals were overall significantly more active than the Control group (*p* < 0.050; Figure 2A). In the same day range, activity increased in all animals during daytime (*p* < 0.001; Figure 2A). Both groups showed increased activity over time between day ranges 1–7 [daytime (*p* < 0.001); nighttime (*p* < 0.001); overall (*p* < 0.001)] and 1–33 (nighttime, *p* < 0.017; Figure 2B). Contrary to nighttime activity, daytime activity decreased between day 1–33 in both groups (*p* < 0.020; Figure 2B).

### Distance

**FIGURE 3:**
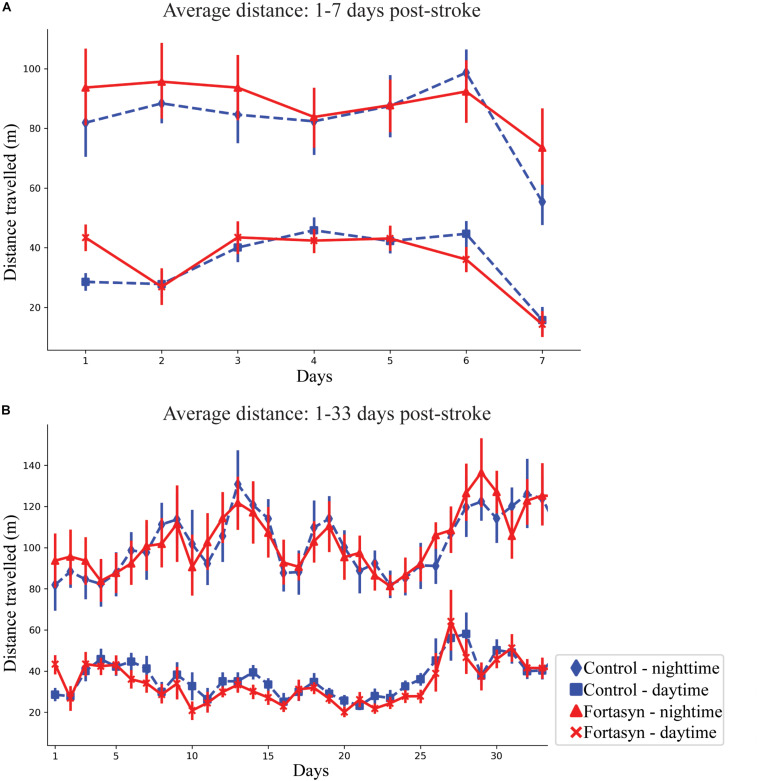
Average distance traveled by Control and Fortasyn fed animals during day- and nighttime between day ranges 1–7 **(A)** and 1–33 **(B)**. **(A,B)** In all animals the distance traveled increased over time during daytime [days: 1–3 (*p* < 0.038), days: 1–7 (*p* < 0.012)], nighttime (days: 1–33 *p* < 0.001), and overall (days: 1–33 *p* < 0.001).

In all animals the average distance traveled was observed to increase during daytime between day ranges 1–3 (*p* < 0.038) and 1–7 (*p* < 0.012; Figure 3A). Between day 1–33 an increase in traveled distance was observed during nighttime (*p* < 0.001) and overall (*p* < 0.001; Figure 3B). No diet effects were found over time.

### Velocity

**FIGURE 4:**
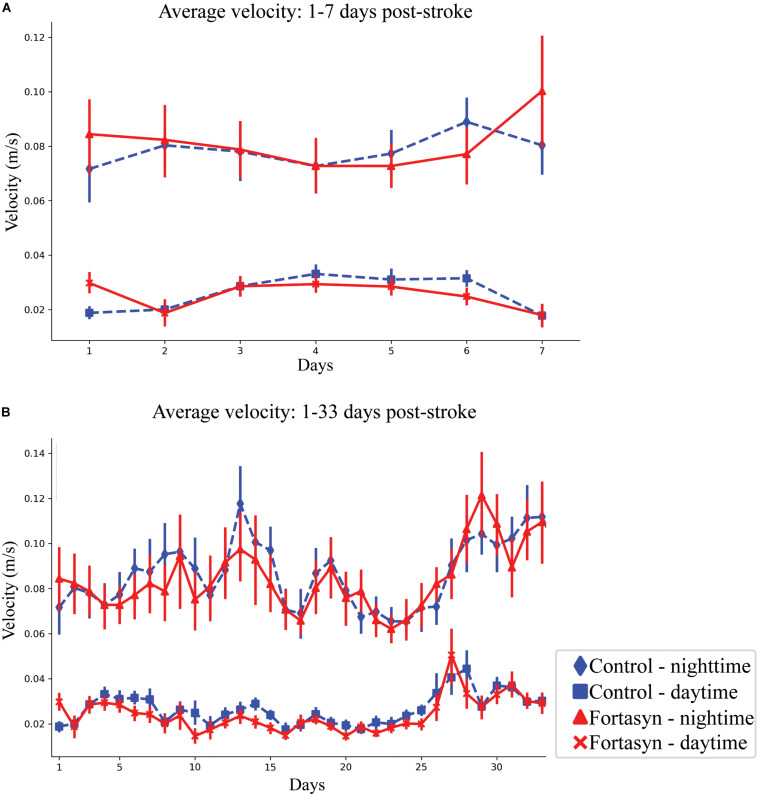
Average velocity measured in Control and Fortasyn fed animals during day- and nighttime between day ranges 1–7 **(A)** and 1–33 **(B)**. **(A)** Average velocity was significantly higher in the Fortasyn group compared to Control during daytime between day ranges 1–3 (*p* < 0.008). **(A,B)** In all animals walking velocity increased over time during daytime [days: 1–3 (*p* < 0.004), days: 1–7 (*p* < 0.001)], nighttime [days: 1–33 (*p* < 0.001)], and overall [days: 1–7 (*p* < 0.059), days: 1–33 (*p* < 0.008)].

All animals displayed increased mean velocity over time during daytime between day ranges 1–3 (*p* < 0.004), 1–7 (*p* < 0.001), 1–33 (*p* < 0.001), and also overall between day range 1–33 (*p* < 0.008; Figure 4B). A diet effect was detected between day 1–3 during daytime (Figure 4A). In detail, Fortasyn fed animals were significantly faster in comparison to the Control group (*p* < 0.008; Figure 4A).

### Turns and Laterality

**FIGURE 5:**
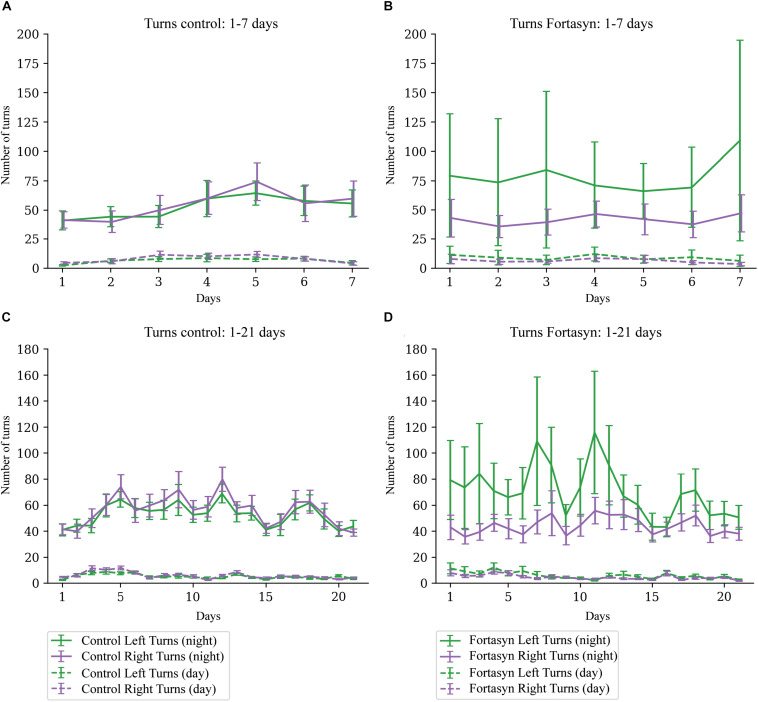
Number of turns measured in Control [**(A)** 1–7 days, **(C)** 1–21 days] and Fortasyn [**(B)** 1–7 days, **(D)** 1–21 days] group during day- and nighttime between different day ranges. No effect of time or diet was detected.

**FIGURE 6:**
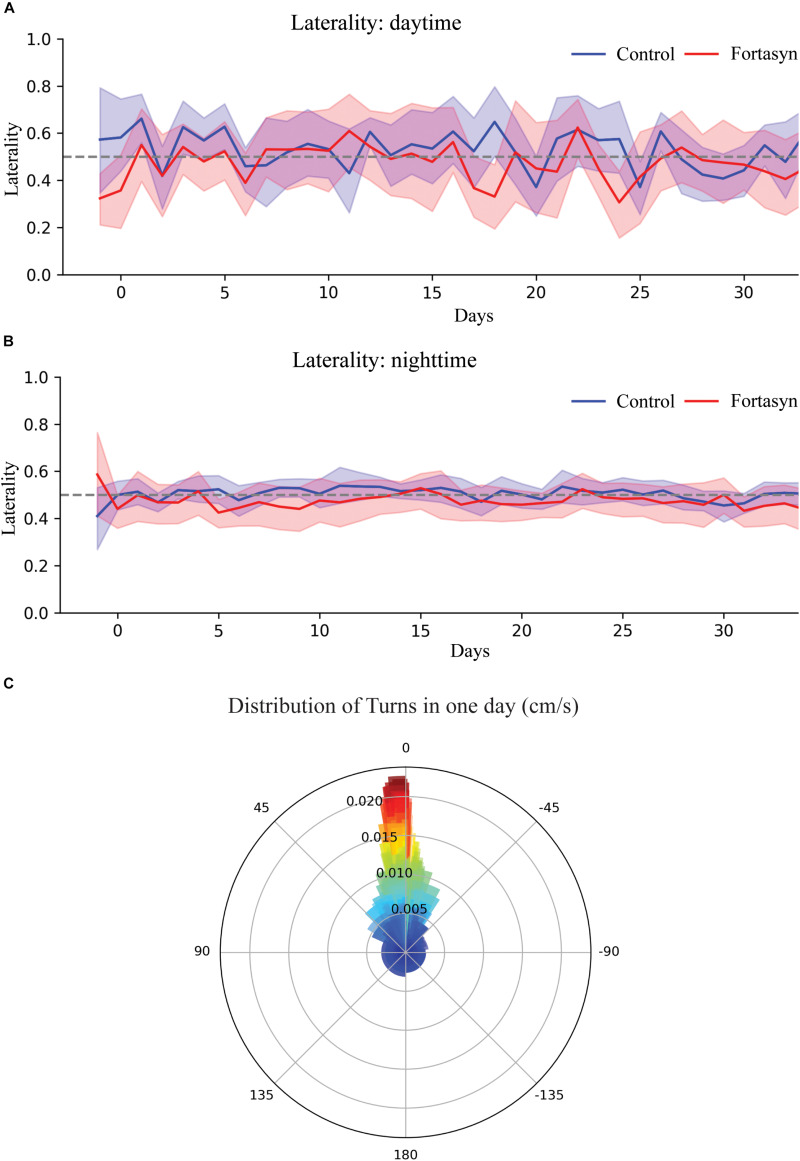
Visualization and analysis of turns. **(A)** Laterality in Fortasyn and Control group during daytime **(A)** and nighttime **(B)** following stroke. **(C)** Polar bar chart of angular movement of one mouse over the period of one day produced by Traja, visualizing laterality.

No significant diet or time differences were found regarding the number of right and left turns during day- and nighttime (Figure 5). Laterality is defined as proportion of right turns over all turns, thus laterality < 0.5 indicates left turn preference (Figures 6A–C). No significant effects due to diet or time were found between groups. In both experimental groups Traja was able to detect left and right turns. Laterality index was observed to be consistently around 0.5, indicating similar number of left and right turns during recovery.

## Discussion

We have demonstrated several capabilities of Traja relevant to behavioral analysis of a stroke mouse model. An increasing number of sensors for animal tracking in recent years has led to a plethora of possibilities to analyze activity, each having advantages and disadvantages (reviewed in Chaumont et al., 2018). Monitoring single housed mice in their home-cages allows to increase the validity of observations for ongoing experiments. The disruption of mouse activity caused by cage changes has been previously documented (Pernold et al., 2019). It is clear that home-cage analysis is useful to monitor interference effects of novel environments. Testing animals in an environment consistent with daily living is thus crucial to maximizing the validity of behavioral assays, and Traja supports analysis of such data.

In the present experimental setting, we investigated the effect of a dietary intervention with Fortasyn acute after stroke induction in female, wildtype C57BL/6JRj mice (Wiesmann et al., 2018). Parameters, including activity, distance, velocity, and laterality were analyzed based on DVC metric measures with Traja to detect differences in recovery between intervention and Control group. Aforementioned parameters were analyzed between different day ranges to explore stroke or diet effects on short term (day range 1–3, 1–7) and long term post-surgery (day range 1–33). Traja calculated a subtle increase in overall activity and daytime velocity in the Fortasyn group compared to the Control group between day 1–3. Previous studies have clearly shown that Fortasyn has neuroprotective effects after an ischemic stroke, however, short-term effects have not been shown before and need to be further investigated (Wiesmann et al., 2017, 2018). Further diet effects were negligible regarding distance, turns and laterality. Both, the Control and Fortasyn group showed progressive recovery from stroke over time. More precisely, in both diet groups Traja calculated an increase of activity, distance, and velocity on short-term and/or long-term after stroke induction. In contrast, activity was overall found to be decreased during daytime (day range 1–33). This lower daytime activity is likely to be the consequence of the many behavioral tests which were performed during daytime on several days during the experiment (detailed overview in Supplementary Figure S2; Wiesmann et al., 2018).

In comparison to standard behavioral tests as the OF, automated analysis of DVC trajectory data with Traja was able to detect differences in walked distance and velocity in the present female stroke animal model during the post-surgery period (Wiesmann et al., 2018). Previously, neither time nor diet differences on locomotion (distance, velocity) have been found in the OF (Wiesmann et al., 2018). In future, further exploration of hyperparameters for laterality (i.e., distance threshold and turn angle range) could improve observation of stroke-induced turn preference, thus potentially providing a quantitative measure of impairment and recovery. In conclusion, the generated data provide a proof-of-concept of Traja as novel automated analysis method of activity measured via home-cage sensors in DVC to quantify several locomotive parameters in a stroke mouse model.

Automated home-cage 24/7 monitoring is an active area of research and a step forward for reproducible behavioral analysis, accompanied by rapid advances in technology and advanced methods such as machine learning (ML). While researchers seek to control many factors in experimental settings to understand biological and pathological processes, there is still much opportunity to extract and analyze large datasets in an experiment. As the amount of data available to researchers grows beyond the capacity of researchers to process and analyze it, tools which support automated pattern recognition are becoming increasingly relevant to neuropsychiatric research and disease treatment. As an open source, Python-based software, Traja supports collaboration between behavioral researchers for both classical hypothesis testing as well as complementary advanced analytical techniques for pattern detection such as unsupervised ML. Based on these findings, we suggest that Traja can be used to gain insight into mouse locomotion in movement disorders and stroke research.

In future, by combining sensor data provided by devices like DVC with open source tools such as Traja, researchers will be able to gain a deeper insight into underlying cognitive processes relevant to neurological conditions like stroke as well as ordinary behavior. Further potential extensions of this software include development of a graphical user interface to increase the usability to researchers with minimal computer skills. Researchers can use tools like Traja to generate highly reproducible and transparent analysis and visualizations of spatial trajectory data collected through virtually any tracking data source.

## Data Availability Statement

The datasets generated for this study are available on request to the corresponding author.

## Ethics Statement

The animal study was reviewed and approved by the Dierexperimentencommissie; DEC (RU−DEC 2014−171).

## Author Contributions

AK and MW provided conceptual guidance and were together with JS and KL involved in the study design. MW collected all the experimental data. JS performed data analysis with Traja as well as statistical analysis. The manuscript was written by JS and KL and commented on by all authors.

## Conflict of Interest

The authors declare that the research was conducted in the absence of any commercial or financial relationships that could be construed as a potential conflict of interest.
