# Supplementary material for: Automated Analysis of Stroke Mouse Trajectory Data With Traja
Source: Front Neurosci. 2020 May 25;14:518. doi: 10.3389/fnins.2020.00518 (PMC7262161; doi:10.3389/fnins.2020.00518)
Supplement: Supplementary file 1 [file Data_Sheet_1.pdf]

Supplementary material

Table 1 A list of functionalities of Traja which were used in the presented study.

**Functionality**	**Description**
traja.step_lengths	Compute step length
traja.calc_displacement	Compute displacement
traja.calc_derivatives	Compute velocity and acceleration
traja.autocorrelation	Compute periodic behavior
traja.calc_heading	Compute heading as angle
traja.calc_turn_angle	Compute turn angle
traja.calc_laterality	Compute laterality as ratio of right turns over all turns, parameterized by minimal distance moved and angle threshold
traja.autocorrelation	Compute periodic behavior
traja.plot_actogram	Plot activity as a line graph
traja.plot_immobility	Plot immobility (sleep), parameterized by time threshold (Figure X)
traja.plot_clustermap	Plot clustered displacement vectors for multiple subjects (Figure X)

Table 2 A list of program items implemented in Traja.

Item Name	***URL Link***
Python 3.6	https://www.python.org/downloads/release/python-360/
pandas 0.24.1	https://pandas.pydata.org/getpandas.html
scipy 1.2.1	https://www.scipy.org/scipylib/download.html
statsmodel 0.9.0	https://www.statsmodels.org/stable/install.html
scikit-learn 0.21.1	https://scikit-learn.org/stable/install.html
PyTorch 0.1.0	https://pytorch.org/get-started/locally/

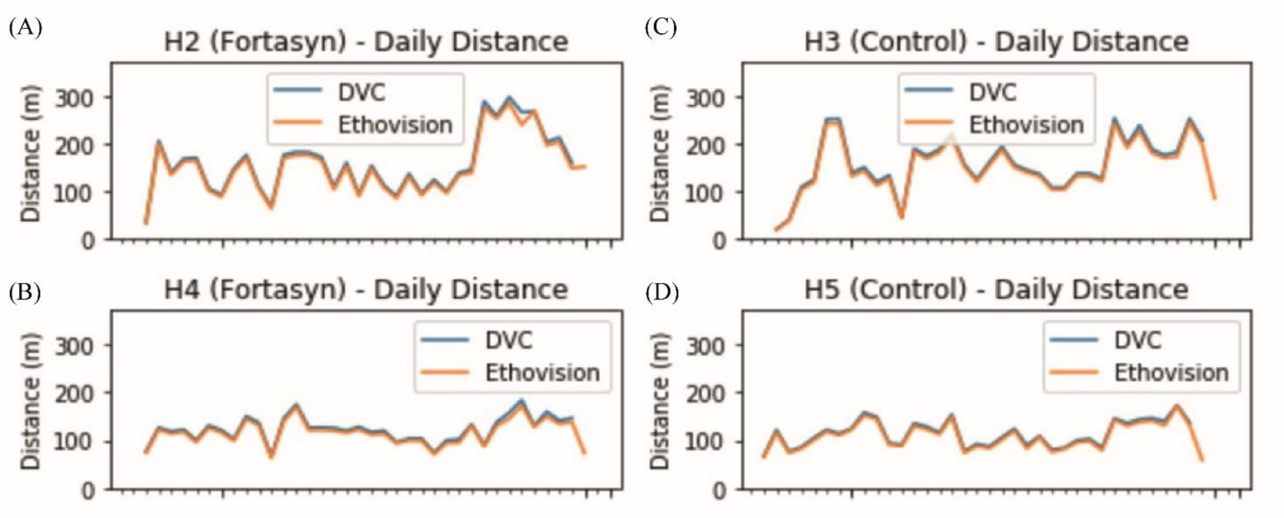

Figure 1 Single digital ventilated cages displaying the walked distance of individual female stroke mice on (A-B) Fortasyn diet and (C-D) Control diet, computed with Traja and Ethovision XT 14 across 33 days. In all 4 cages the calculated distances are matching.

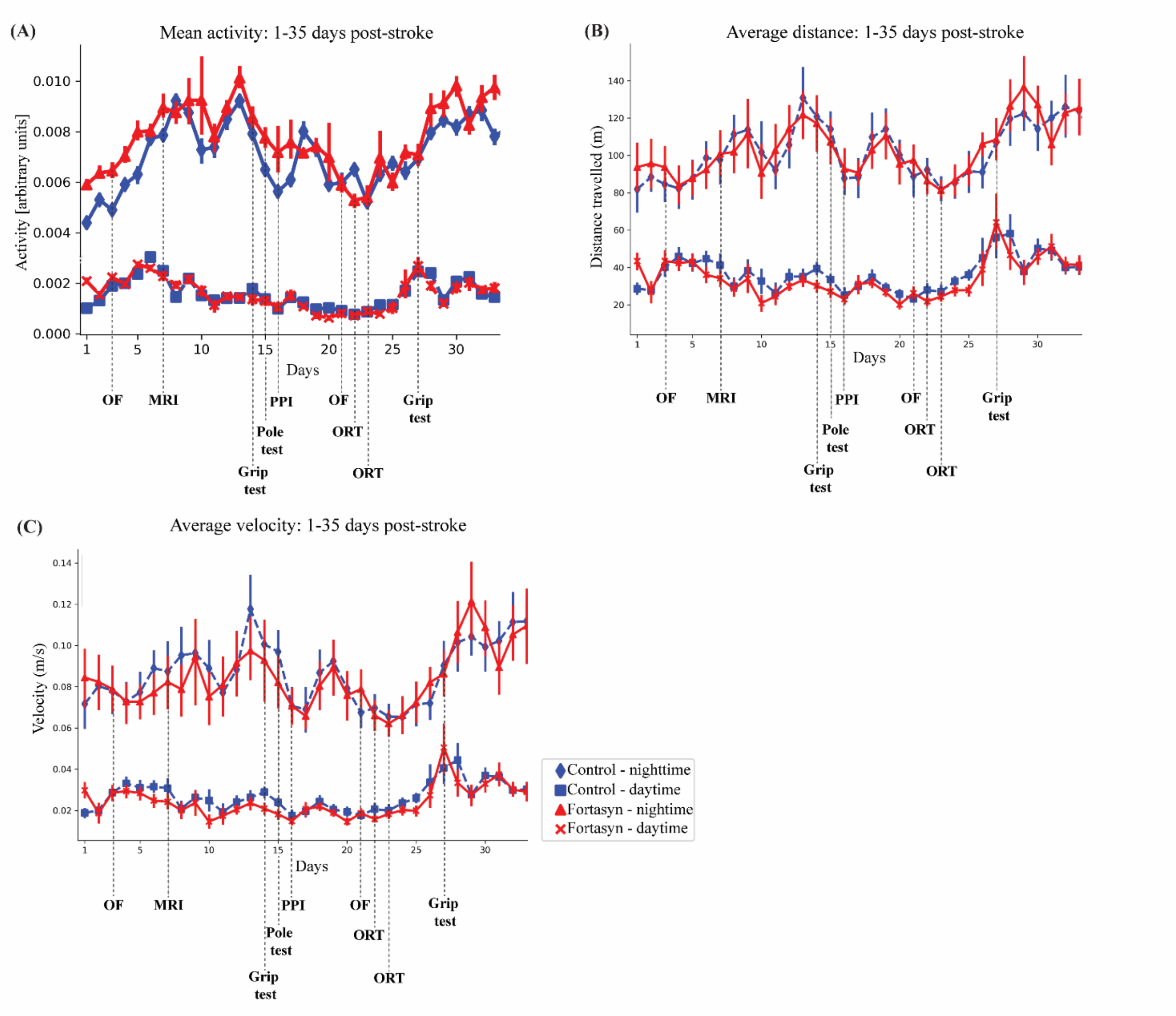

Figure 2 The figures are showing the day- and nighttime activity (A), distance travelled (B), and velocity (C) of Control and Fortasyn animals over 33 days. All performed behavioral tests are highlighted, including the Open Field test (OF) on day 3 and 21, MRI (Magnetic resonance imaging) on day 7 and 35, Grip test on day 14 and 27, Pole test on day 15, Prepulse inhibition test (PPI) on day 16, and Object recognition test (ORT) on day 22 and 23.

Statistical tables

Displacement (Days 1-3)

```
Dark GLM RESULTS:
                               GEE Regression Results
    ===================================================================================
    Dep. Variable:               displacement   No. Observations:                   69
    Model:                                GEE   No. clusters:                       23
    Method:                       Generalized   Min. cluster size:                   3
                         Estimating Equations   Max. cluster size:                   3
    Family:                          Gaussian   Mean cluster size:                 3.0
    Dependence structure:        Independence   Num. iterations:                     2
    Date:                    Thu, 11 Apr 2019   Scale:                        1468.203
    Covariance type:                   robust   Time:                         14:54:32
    =====================================================================================
                            coef    std err          z      P>|z|      [0.025      0.975]
    -------------------------------------------------------------------------------------
    Intercept            91.1269     14.098      6.464      0.000      63.496     118.757
    diet[T.Fortasyn]     10.1586     10.228      0.993      0.321      -9.888      30.205
    days_from_surgery     0.8113      6.096      0.133      0.894     -11.137      12.759
    ==============================================================================
    Skew:                          0.4587   Kurtosis:                         0.4001
    Centered skew:                 0.6235   Centered kurtosis:                0.3274
    ==============================================================================
    END Dark GLM RESULTS
```

```
    Light GLM RESULTS:
                               GEE Regression Results
    ===================================================================================
    Dep. Variable:               displacement   No. Observations:                   69
    Model:                                GEE   No. clusters:                       23
    Method:                       Generalized   Min. cluster size:                   3
                         Estimating Equations   Max. cluster size:                   3
    Family:                          Gaussian   Mean cluster size:                 3.0
    Dependence structure:        Independence   Num. iterations:                     2
    Date:                    Thu, 11 Apr 2019   Scale:                         209.368
    Covariance type:                   robust   Time:                         14:54:32
    =====================================================================================
                            coef    std err          z      P>|z|      [0.025      0.975]
    -------------------------------------------------------------------------------------
    Intercept            18.5143      3.156      5.866      0.000      12.329      24.700
```

```
diet[T.Fortasyn]      4.9762      4.833      1.030      0.303      -4.496      14.449
days_from_surgery     2.8843      1.393      2.070      0.038       0.154       5.615
==============================================================================
Skew:                         0.7917   Kurtosis:                         0.3263
Centered skew:               -0.2495   Centered kurtosis:                0.3249
==============================================================================
days_from_surgery effect on displacement (Light) - p-value: 0.0384
END Light GLM RESULTS
```

```
Overall GLM RESULTS:
                               GEE Regression Results
===================================================================================
Dep. Variable:               displacement   No. Observations:                    69
Model:                                GEE   No. clusters:                        23
Method:                       Generalized   Min. cluster size:                    3
                      Estimating Equations   Max. cluster size:                    3
Family:                          Gaussian   Mean cluster size:                  3.0
Dependence structure:        Independence   Num. iterations:                      2
Date:                    Thu, 11 Apr 2019   Scale:                         1984.162
Covariance type:                   robust   Time:                          14:54:32
=====================================================================================
                        coef    std err          z      P>|z|      [0.025      0.975]
-------------------------------------------------------------------------------------
Intercept           109.6412     15.277      7.177      0.000      79.698     139.584
diet[T.Fortasyn]     15.1349     13.523      1.119      0.263     -11.370      41.640
days_from_surgery     3.6956      6.381      0.579      0.563      -8.812      16.203
==============================================================================
Skew:                         0.1968   Kurtosis:                         0.1147
Centered skew:                0.6899   Centered kurtosis:                0.7732
==============================================================================
END Overall GLM RESULTS
```

Displacement (Days 1-7)

```
Fitting generalized linear model on daily data. Days 1-7
Model: displacement ~ days_from_surgery + diet.
Dark GLM RESULTS:
                               GEE Regression Results
===================================================================================
```

```
Dep. Variable:              displacement   No. Observations:                  161
Model:                               GEE   No. clusters:                       23
Method:                      Generalized   Min. cluster size:                   7
                    Estimating Equations   Max. cluster size:                   7
Family:                         Gaussian   Mean cluster size:                 7.0
Dependence structure:       Independence   Num. iterations:                     2
Date:                   Thu, 11 Apr 2019   Scale:                        1442.049
Covariance type:                  robust   Time:                         14:52:50
=====================================================================================
                        coef    std err          z      P>|z|      [0.025      0.975]
-------------------------------------------------------------------------------------
Intercept            91.9111      5.895     15.592      0.000      80.357     103.465
diet[T.Fortasyn]      3.4535      9.002      0.384      0.701     -14.191      21.098
days_from_surgery     1.4633      1.361      1.075      0.282      -1.203       4.130
==============================================================================
Skew:                          0.9751   Kurtosis:                         1.3637
Centered skew:                 0.6546   Centered kurtosis:                1.1815
==============================================================================
END Dark GLM RESULTS
```

```
Light GLM RESULTS:
                               GEE Regression Results
===================================================================================
Dep. Variable:              displacement   No. Observations:                  161
Model:                               GEE   No. clusters:                       23
Method:                      Generalized   Min. cluster size:                   7
                    Estimating Equations   Max. cluster size:                   7
Family:                         Gaussian   Mean cluster size:                 7.0
Dependence structure:       Independence   Num. iterations:                     2
Date:                   Thu, 11 Apr 2019   Scale:                         176.343
Covariance type:                  robust   Time:                         14:52:50
=====================================================================================
                        coef    std err          z      P>|z|      [0.025      0.975]
-------------------------------------------------------------------------------------
Intercept            24.6560      2.419     10.194      0.000      19.916      29.396
diet[T.Fortasyn]      0.2083      3.274      0.064      0.949      -6.209       6.625
days_from_surgery     1.2145      0.483      2.517      0.012       0.269       2.160
==============================================================================
Skew:                          0.6127   Kurtosis:                         0.1457
Centered skew:                 0.2553   Centered kurtosis:               -0.0809
==============================================================================
days_from_surgery effect on displacement (Light) - p-value: 0.0118
```

```
END Light GLM RESULTS
```

```
Overall GLM RESULTS:
                               GEE Regression Results
===================================================================================
Dep. Variable:                displacement   No. Observations:                  161
Model:                                 GEE   No. clusters:                       23
Method:                        Generalized   Min. cluster size:                   7
                      Estimating Equations   Max. cluster size:                   7
Family:                           Gaussian   Mean cluster size:                 7.0
Dependence structure:         Independence   Num. iterations:                     2
Date:                     Thu, 11 Apr 2019   Scale:                        1906.170
Covariance type:                    robust   Time:                         14:52:50
=====================================================================================
                        coef    std err          z      P>|z|      [0.025      0.975]
-------------------------------------------------------------------------------------
Intercept           116.5671      7.219     16.147      0.000     102.418     130.716
diet[T.Fortasyn]      3.6618     11.439      0.320      0.749     -18.759      26.082
days_from_surgery     2.6778      1.595      1.679      0.093      -0.448       5.804
==============================================================================
Skew:                          0.7669   Kurtosis:                         0.9762
Centered skew:                 0.4431   Centered kurtosis:                1.1477
==============================================================================
END Overall GLM RESULTS
```

Displacement (Days 1-33)

```
Fitting generalized linear model on daily data. Days 1-33
Model: displacement ~ days_from_surgery + diet.
Dark GLM RESULTS:
                               GEE Regression Results
===================================================================================
Dep. Variable:                displacement   No. Observations:                  759
Model:                                 GEE   No. clusters:                       23
Method:                        Generalized   Min. cluster size:                  32
                      Estimating Equations   Max. cluster size:                  34
Family:                           Gaussian   Mean cluster size:                33.0
Dependence structure:         Independence   Num. iterations:                     2
Date:                     Thu, 11 Apr 2019   Scale:                        1801.456
Covariance type:                    robust   Time:                         14:55:10
```

```
==================================================================================
                        coef    std err          z      P>|z|      [0.025      0.975]
----------------------------------------------------------------------------------
Intercept            98.5194      5.589     17.628      0.000      87.565     109.473
diet[T.Fortasyn]      0.5593     10.582      0.053      0.958     -20.182      21.300
days_from_surgery     0.9200      0.161      5.709      0.000       0.604       1.236
==============================================================================
Skew:                          0.8281   Kurtosis:                         1.4631
Centered skew:                 0.2984   Centered kurtosis:                1.1118
==============================================================================
days_from_surgery effect on displacement (Dark) - p-value: 0.0000
END Dark GLM RESULTS
```

```
Light GLM RESULTS:
                               GEE Regression Results
===================================================================================
Dep. Variable:                displacement   No. Observations:                  757
Model:                                 GEE   No. clusters:                       23
Method:                        Generalized   Min. cluster size:                  32
                      Estimating Equations   Max. cluster size:                  33
Family:                           Gaussian   Mean cluster size:                32.9
Dependence structure:         Independence   Num. iterations:                     2
Date:                     Thu, 11 Apr 2019   Scale:                         272.419
Covariance type:                    robust   Time:                         14:55:10
==================================================================================
                        coef    std err          z      P>|z|      [0.025      0.975]
----------------------------------------------------------------------------------
Intercept            24.8763      1.648     15.094      0.000      21.646      28.107
diet[T.Fortasyn]     -2.3441      2.019     -1.161      0.246      -6.302       1.614
days_from_surgery    -0.0085      0.055     -0.153      0.878      -0.117       0.100
==============================================================================
Skew:                          2.3559   Kurtosis:                         8.5764
Centered skew:                 2.4101   Centered kurtosis:                9.3801
==============================================================================
END Light GLM RESULTS
```

```
Overall GLM RESULTS:
                               GEE Regression Results
```

```
==================================================================================
Dep. Variable:              displacement   No. Observations:                   757
Model:                               GEE   No. clusters:                        23
Method:                      Generalized   Min. cluster size:                   32
                    Estimating Equations   Max. cluster size:                   33
Family:                         Gaussian   Mean cluster size:                 32.9
Dependence structure:       Independence   Num. iterations:                      2
Date:                   Thu, 11 Apr 2019   Scale:                         2226.498
Covariance type:                  robust   Time:                          14:55:10
====================================================================================
                        coef    std err          z      P>|z|      [0.025      0.975]
------------------------------------------------------------------------------------
Intercept           123.1486      5.936     20.748      0.000     111.515     134.782
diet[T.Fortasyn]     -1.7406     12.023     -0.145      0.885     -25.305      21.824
days_from_surgery     0.9438      0.168      5.617      0.000       0.614       1.273
==============================================================================
Skew:                          0.8755   Kurtosis:                          1.5004
Centered skew:                 0.3315   Centered kurtosis:                 0.7931
==============================================================================
days_from_surgery effect on displacement (Overall) - p-value: 0.0000
END Overall GLM RESULTS
```

Velocity (Days 1-3)

```
Nighttime GLM RESULTS:
                    GEE Regression Results
==================================================================================
Dep. Variable:                velocity No. Observations:                 69
Model:                             GEE No. clusters:   23
Method:                    Generalized Min. cluster size:                   3
                      Estimating Equations Max. cluster size:              3
Family:                       Gaussian Mean cluster size:            3.0
Dependence structure:         Independence Num. iterations:                 2
Date:                     Tue, 14 Jan 2020 Scale:                        648.891
Covariance type:                    robust Time:   23:48:25
==========================================================================================
                  coef    std err         z P>|z| [0.025      0.975]
------------------------------------------------------------------------------------------
Intercept                46.4065 11.216 4.138      0.000 24.423 68.389
diet[T.Fortasyn]         11.3562 18.189 0.624      0.532 -24.294 47.007
days                      2.1031 5.149 0.408      0.683 -7.989 12.196
days:diet[T.Fortasyn]    -3.9696 7.533 -0.527      0.598 -18.735 10.796
==============================================================================
```

```
Skew:                          0.8330 Kurtosis:          0.7739
Centered skew:                 0.6436 Centered kurtosis:          0.5974
==============================================================================
END Nighttime GLM RESULTS
```

```
Daytime GLM RESULTS:
                    GEE Regression Results
===================================================================================
Dep. Variable:                    velocity No. Observations:                  69
Model:                                 GEE No. clusters:   23
Method:                        Generalized Min. cluster size:                  3
                      Estimating Equations Max. cluster size:              3
Family:                           Gaussian Mean cluster size:            3.0
Dependence structure:         Independence Num. iterations:                    2
Date:                     Tue, 14 Jan 2020 Scale:                        83.255
Covariance type:                    robust Time:   23:48:25
=========================================================================================
                 coef    std err         z P>|z| [0.025      0.975]
-----------------------------------------------------------------------------------------
Intercept                 9.8937 2.452 4.035      0.000 5.088 14.700
diet[T.Fortasyn]         11.0901 4.207 2.636      0.008 2.845 19.335
days                      3.8247 1.318 2.902      0.004 1.242 6.408
days:diet[T.Fortasyn]    -4.2952 1.736 -2.474      0.013 -7.698 -0.892
==============================================================================
Skew:                          0.6217 Kurtosis:          0.6256
Centered skew:                -0.0003 Centered kurtosis:          -0.1508
==============================================================================
diet[T.Fortasyn] effect on velocity (Daytime) - p-value: 0.0084
days effect on velocity (Daytime) - p-value: 0.0037
days:diet[T.Fortasyn] effect on velocity (Daytime) - p-value: 0.0134
END Daytime GLM RESULTS
```

```
Overall GLM RESULTS:
                    GEE Regression Results
===================================================================================
Dep. Variable:                    velocity No. Observations:                  69
Model:                                 GEE No. clusters:   23
Method:                        Generalized Min. cluster size:                  3
              Estimating Equations   Max. cluster size:              3
Family:                           Gaussian Mean cluster size:            3.0
Dependence structure:         Independence Num. iterations:                    2
Date:                     Tue, 14 Jan 2020 Scale:                         947.699
```

```
Covariance type:                    robust Time:   23:48:25
==========================================================================================
                  coef    std err         z P>|z| [0.025      0.975]
------------------------------------------------------------------------------------------
Intercept                56.3002 12.838 4.385      0.000 31.138 81.463
diet[T.Fortasyn]         22.4463 21.624 1.038      0.299 -19.936 64.829
days                      5.9278 5.870 1.010      0.313 -5.577 17.432
days:diet[T.Fortasyn]    -8.2648 8.579 -0.963      0.335 -25.079 8.549
===============================================================================
Skew:                          0.6685 Kurtosis:           0.4350
Centered skew:                 0.7563 Centered kurtosis:           0.8609
===============================================================================
END Overall GLM RESULTS
```

Velocity (Days 1-7)

```
Nighttime GLM RESULTS:
                    GEE Regression Results
================================================================================
Dep. Variable:                    velocity No. Observations:                 161
Model:                                 GEE No. clusters:   23
Method:                        Generalized Min. cluster size:                  7
                      Estimating Equations Max. cluster size:              7
Family:                           Gaussian Mean cluster size:            7.0
Dependence structure:         Independence Num. iterations:                    2
Date:                     Tue, 14 Jan 2020 Scale:                        592.866
Covariance type:                    robust Time:   23:48:25
==========================================================================================
                  coef    std err         z P>|z| [0.025      0.975]
------------------------------------------------------------------------------------------
Intercept                46.4478 4.936 9.410      0.000 36.774 56.122
diet[T.Fortasyn]          7.6137 9.019 0.844      0.399 -10.064 25.291
days                      1.5062 1.418 1.062      0.288 -1.274 4.286
days:diet[T.Fortasyn]    -2.0437 1.910 -1.070      0.285 -5.787 1.700
===============================================================================
Skew:                          1.2133 Kurtosis:           2.0659
Centered skew:                 0.6824 Centered kurtosis:           1.2058
===============================================================================
END Nighttime GLM RESULTS
```

```
Daytime GLM RESULTS:
                    GEE Regression Results
```

```
==============================================================================
Dep. Variable:                  velocity No. Observations:                  161
Model:                               GEE No. clusters:   23
Method:                      Generalized Min. cluster size:                   7
                      Estimating Equations Max. cluster size:              7
Family:                         Gaussian Mean cluster size:            7.0
Dependence structure:         Independence Num. iterations:                   2
Date:                    Tue, 14 Jan 2020 Scale:                         80.200
Covariance type:                  robust Time:   23:48:25
==========================================================================================
                 coef    std err         z P>|z| [0.025      0.975]
------------------------------------------------------------------------------------------
Intercept               14.7656 1.558 9.479      0.000 11.712 17.819
diet[T.Fortasyn]         6.2525 3.388 1.845      0.065 -0.388 12.893
days                     1.7138 0.426 4.026      0.000 0.879 2.548
days:diet[T.Fortasyn]    -1.8449 0.586 -3.148      0.002 -2.993 -0.696
==============================================================================
Skew:                          0.4130 Kurtosis:         -0.1300
Centered skew:                 0.0573 Centered kurtosis:         -0.1385
==============================================================================
days effect on velocity (Daytime) - p-value: 0.0001
days:diet[T.Fortasyn] effect on velocity (Daytime) - p-value: 0.0016
END Daytime GLM RESULTS
```

```
Overall GLM RESULTS:
                    GEE Regression Results
==============================================================================
Dep. Variable:                  velocity No. Observations:                  161
Model:                               GEE No. clusters:   23
Method:                      Generalized Min. cluster size:                   7
              Estimating Equations   Max. cluster size:              7
Family:                         Gaussian Mean cluster size:            7.0
Dependence structure:         Independence Num. iterations:                   2
Date:                    Tue, 14 Jan 2020 Scale:                         861.084
Covariance type:                  robust Time:   23:48:25
==========================================================================================
                 coef    std err         z P>|z| [0.025      0.975]
------------------------------------------------------------------------------------------
Intercept               61.2134 6.102 10.032      0.000 49.254 73.173
diet[T.Fortasyn]        13.8663 11.049 1.255      0.209 -7.789 35.522
days                     3.2200 1.705 1.889      0.059 -0.122 6.562
days:diet[T.Fortasyn]    -3.8886 2.134 -1.822      0.068 -8.072 0.295
==============================================================================
Skew:                          1.0544 Kurtosis:          1.7142
Centered skew:                 0.5942 Centered kurtosis:          1.1729
```

```
==============================================================================
END Overall GLM RESULTS
```

Velocity (Days 1-33)

```
Fitting generalized linear model on daily data. Days 1-33
Model: velocity ~ days_from_surgery * diet.
Nighttime GLM RESULTS:
                    GEE Regression Results
===================================================================================
Dep. Variable:                  velocity No. Observations:                 759
Model:                               GEE No. clusters:   23
Method:                      Generalized Min. cluster size:                 33
                      Estimating Equations Max. cluster size:             33
Family:                         Gaussian Mean cluster size:           33.0
Dependence structure:        Independence Num. iterations:                   2
Date:                    Tue, 14 Jan 2020 Scale:                       732.344
Covariance type:                   robust Time:   23:48:25
=========================================================================================
                  coef    std err         z P>|z| [0.025      0.975]
-----------------------------------------------------------------------------------------
Intercept               52.1261 3.459 15.072      0.000 45.348 58.905
diet[T.Fortasyn]        -3.0980 6.803 -0.455      0.649 -16.431 10.235
days                     0.2842 0.168 1.690      0.091 -0.045 0.614
days:diet[T.Fortasyn]    0.1030 0.201 0.513      0.608 -0.291 0.497
==============================================================================
Skew:                       1.4313 Kurtosis:           2.9769
Centered skew:              0.7356 Centered kurtosis:           1.6847
==============================================================================
END Nighttime GLM RESULTS
```

```
Daytime GLM RESULTS:
                    GEE Regression Results
===================================================================================
Dep. Variable:                  velocity No. Observations:                 759
Model:                               GEE No. clusters:   23
Method:                      Generalized Min. cluster size:                 33
              Estimating Equations   Max. cluster size:             33
Family:                         Gaussian Mean cluster size:           33.0
Dependence structure:        Independence Num. iterations:                   2
Date:                    Tue, 14 Jan 2020 Scale:                       131.084
Covariance type:                   robust Time:   23:48:25
=========================================================================================
```

```
                 coef    std err         z P>|z| [0.025      0.975]
------------------------------------------------------------------------------------------
Intercept               17.5764 1.194 14.716      0.000 15.235 19.917
diet[T.Fortasyn]        -1.9007 2.084 -0.912      0.362 -5.986 2.185
days                     0.1858 0.057 3.286      0.001 0.075 0.297
days:diet[T.Fortasyn]   -0.0024 0.092 -0.026      0.979 -0.182 0.177
==============================================================================
Skew:                          2.2375 Kurtosis:          9.8639
Centered skew:                 2.1180 Centered kurtosis:           9.9868
==============================================================================
days effect on velocity (Daytime) - p-value: 0.0010
END Daytime GLM RESULTS
```

```
Overall GLM RESULTS:
                    GEE Regression Results
====================================================================================
Dep. Variable:                  velocity No. Observations:                 759
Model:                               GEE No. clusters:   23
Method:                      Generalized Min. cluster size:                 33
                      Estimating Equations Max. cluster size:             33
Family:                         Gaussian Mean cluster size:           33.0
Dependence structure:         Independence Num. iterations:                  2
Date:                     Tue, 14 Jan 2020 Scale:                      1090.772
Covariance type:                  robust Time:   23:48:25
==========================================================================================
                 coef    std err         z P>|z| [0.025      0.975]
------------------------------------------------------------------------------------------
Intercept               69.7025 3.833 18.185      0.000 62.190 77.215
diet[T.Fortasyn]        -4.9987 8.251 -0.606      0.545 -21.170 11.173
days                     0.4700 0.177 2.655      0.008 0.123 0.817
days:diet[T.Fortasyn]    0.1005 0.226 0.445      0.657 -0.343 0.544
==============================================================================
Skew:                          1.3130 Kurtosis:          2.6132
Centered skew:                 0.5748 Centered kurtosis:          1.4120
==============================================================================
days effect on velocity (Overall) - p-value: 0.0079
END Overall GLM RESULTS
```

Activity (Days 1-3)

Model: activity ~ days_from_surgery + diet.

```
Dark GLM RESULTS:
```

```
                               GEE Regression Results
===================================================================================
Dep. Variable:                     activity   No. Observations:                  69
Model:                                  GEE   No. clusters:                      23
Method:                         Generalized   Min. cluster size:                  3
                      Estimating Equations   Max. cluster size:                  3
Family:                            Gaussian   Mean cluster size:                3.0
Dependence structure:          Independence   Num. iterations:                    2
Date:                      Thu, 11 Apr 2019   Scale:                          0.000
Covariance type:                     robust   Time:                        16:33:18
=====================================================================================
                        coef    std err          z      P>|z|      [0.025      0.975]
-------------------------------------------------------------------------------------
Intercept             0.0042      0.001      4.929      0.000       0.003       0.006
diet[T.Fortasyn]      0.0013      0.001      1.860      0.063   -6.96e-05       0.003
days_from_surgery     0.0003      0.000      0.996      0.319      -0.000       0.001
==============================================================================
Skew:                          0.0608   Kurtosis:                        -0.0946
Centered skew:                 0.4392   Centered kurtosis:               -0.2021
==============================================================================
END Dark GLM RESULTS
```

```
Light GLM RESULTS:
                               GEE Regression Results
===================================================================================
Dep. Variable:                     activity   No. Observations:                  69
Model:                                  GEE   No. clusters:                      23
Method:                         Generalized   Min. cluster size:                  3
                      Estimating Equations   Max. cluster size:                  3
Family:                            Gaussian   Mean cluster size:                3.0
Dependence structure:          Independence   Num. iterations:                    2
Date:                      Thu, 11 Apr 2019   Scale:                          0.000
Covariance type:                     robust   Time:                        16:33:18
=====================================================================================
                        coef    std err          z      P>|z|      [0.025      0.975]
-------------------------------------------------------------------------------------
Intercept             0.0008      0.000      4.145      0.000       0.000       0.001
diet[T.Fortasyn]      0.0005      0.000      1.776      0.076   -5.22e-05       0.001
days_from_surgery     0.0003   8.72e-05      3.175      0.001       0.000       0.000
==============================================================================
Skew:                          0.3639   Kurtosis:                        -0.3670
Centered skew:                 0.0021   Centered kurtosis:                0.3980
```

```
==============================================================================
days_from_surgery effect on activity (Light) - p-value: 0.0015
END Light GLM RESULTS
```

```
Overall GLM RESULTS:
                               GEE Regression Results
===================================================================================
Dep. Variable:                     activity   No. Observations:                   69
Model:                                  GEE   No. clusters:                       23
Method:                         Generalized   Min. cluster size:                   3
                      Estimating Equations   Max. cluster size:                   3
Family:                            Gaussian   Mean cluster size:                 3.0
Dependence structure:          Independence   Num. iterations:                     2
Date:                      Thu, 11 Apr 2019   Scale:                           0.000
Covariance type:                     robust   Time:                         16:33:18
=====================================================================================
                        coef    std err          z      P>|z|      [0.025      0.975]
-------------------------------------------------------------------------------------
Intercept             0.0025      0.000      5.123      0.000       0.002       0.003
diet[T.Fortasyn]      0.0009      0.000      1.960      0.050     2.6e-08       0.002
days_from_surgery     0.0003      0.000      1.702      0.089   -4.38e-05       0.001
==============================================================================
Skew:                         -0.0860   Kurtosis:                      -0.3211
Centered skew:                 0.3446   Centered kurtosis:             -0.0814
==============================================================================
diet[T.Fortasyn] effect on activity (Overall) - p-value: 0.0500
END Overall GLM RESULTS
```

Activity (Days 1-7)

```
Dark GLM RESULTS:
                               GEE Regression Results
===================================================================================
Dep. Variable:                     activity   No. Observations:                  161
Model:                                  GEE   No. clusters:                       23
Method:                         Generalized   Min. cluster size:                   7
                      Estimating Equations   Max. cluster size:                   7
Family:                            Gaussian   Mean cluster size:                 7.0
Dependence structure:          Independence   Num. iterations:                     2
Date:                      Thu, 11 Apr 2019   Scale:                           0.000
```

```
Covariance type:                   robust   Time:                         16:34:01
==================================================================================
                         coef    std err          z      P>|z|      [0.025      0.975]
----------------------------------------------------------------------------------
Intercept              0.0040      0.000      8.134      0.000       0.003       0.005
diet[T.Fortasyn]       0.0008      0.001      1.103      0.270      -0.001       0.002
days_from_surgery      0.0005   9.14e-05      5.856      0.000       0.000       0.001
==============================================================================
Skew:                          0.9049   Kurtosis:                         1.4701
Centered skew:                 0.6877   Centered kurtosis:                2.2568
==============================================================================
days_from_surgery effect on activity (Dark) - p-value: 0.0000
END Dark GLM RESULTS
```

```
Light GLM RESULTS:
                               GEE Regression Results
================================================================================
Dep. Variable:                   activity   No. Observations:                  161
Model:                                GEE   No. clusters:                       23
Method:                       Generalized   Min. cluster size:                   7
                      Estimating Equations   Max. cluster size:                   7
Family:                          Gaussian   Mean cluster size:                 7.0
Dependence structure:        Independence   Num. iterations:                     2
Date:                    Thu, 11 Apr 2019   Scale:                           0.000
Covariance type:                   robust   Time:                         16:34:01
==================================================================================
                         coef    std err          z      P>|z|      [0.025      0.975]
----------------------------------------------------------------------------------
Intercept              0.0012      0.000      6.775      0.000       0.001       0.002
diet[T.Fortasyn]       0.0001      0.000      0.565      0.572      -0.000       0.001
days_from_surgery      0.0002   3.58e-05      5.533      0.000       0.000       0.000
==============================================================================
Skew:                          0.4925   Kurtosis:                         0.0648
Centered skew:                 0.0288   Centered kurtosis:                0.6171
==============================================================================
days_from_surgery effect on activity (Light) - p-value: 0.0000
END Light GLM RESULTS
```

```
Overall GLM RESULTS:
                               GEE Regression Results
===================================================================================
Dep. Variable:                     activity   No. Observations:                  161
Model:                                  GEE   No. clusters:                       23
Method:                         Generalized   Min. cluster size:                   7
                      Estimating Equations   Max. cluster size:                   7
Family:                            Gaussian   Mean cluster size:                 7.0
Dependence structure:          Independence   Num. iterations:                     2
Date:                      Thu, 11 Apr 2019   Scale:                           0.000
Covariance type:                     robust   Time:                         16:34:01
=====================================================================================
                       coef    std err          z      P>|z|      [0.025      0.975]
-------------------------------------------------------------------------------------
Intercept            0.0026      0.000      8.387      0.000       0.002       0.003
diet[T.Fortasyn]     0.0005      0.000      0.998      0.318      -0.000       0.001
days_from_surgery    0.0004    5.5e-05      6.670      0.000       0.000       0.000
==============================================================================
Skew:                          0.7503   Kurtosis:                         1.3065
Centered skew:                 0.5865   Centered kurtosis:                2.1950
==============================================================================
days_from_surgery effect on activity (Overall) - p-value: 0.0000
END Overall GLM RESULTS
```

Activity (Days 1-33)

```
Dark GLM RESULTS:
                               GEE Regression Results
===================================================================================
Dep. Variable:                     activity   No. Observations:                  759
Model:                                  GEE   No. clusters:                       23
Method:                         Generalized   Min. cluster size:                  33
                      Estimating Equations   Max. cluster size:                  33
Family:                            Gaussian   Mean cluster size:                33.0
Dependence structure:          Independence   Num. iterations:                     2
Date:                      Thu, 11 Apr 2019   Scale:                           0.000
Covariance type:                     robust   Time:                         16:20:30
=====================================================================================
                       coef    std err          z      P>|z|      [0.025      0.975]
-------------------------------------------------------------------------------------
Intercept            0.0066      0.001     11.396      0.000       0.005       0.008
diet[T.Fortasyn]     0.0003      0.001      0.367      0.713      -0.001       0.002
days_from_surgery  3.35e-05    1.4e-05      2.389      0.017    6.02e-06     6.1e-05
```

```
==============================================================================
Skew:                          0.9077   Kurtosis:                       1.2141
Centered skew:                 0.7236   Centered kurtosis:              1.1281
==============================================================================
days_from_surgery effect on activity (Dark) - p-value: 0.0169
END Dark GLM RESULTS
```

```
Light GLM RESULTS:
                               GEE Regression Results
===================================================================================
Dep. Variable:                   activity   No. Observations:                  759
Model:                                GEE   No. clusters:                       23
Method:                       Generalized   Min. cluster size:                  33
                      Estimating Equations   Max. cluster size:                  33
Family:                          Gaussian   Mean cluster size:                33.0
Dependence structure:         Independence   Num. iterations:                     2
Date:                    Thu, 11 Apr 2019   Scale:                           0.000
Covariance type:                   robust   Time:                         16:20:30
=====================================================================================
                        coef    std err          z      P>|z|      [0.025      0.975]
-------------------------------------------------------------------------------------
Intercept             0.0018      0.000     10.873      0.000       0.002       0.002
diet[T.Fortasyn]  -8.674e-05      0.000     -0.506      0.613      -0.000       0.000
days_from_surgery -1.244e-05   5.35e-06     -2.325      0.020   -2.29e-05   -1.95e-06
==============================================================================
Skew:                          1.7307   Kurtosis:                       4.9171
Centered skew:                 1.6676   Centered kurtosis:              5.1940
==============================================================================
days_from_surgery effect on activity (Light) - p-value: 0.0201
END Light GLM RESULTS
```

```
Overall GLM RESULTS:
                               GEE Regression Results
===================================================================================
Dep. Variable:                   activity   No. Observations:                  759
Model:                                GEE   No. clusters:                       23
Method:                       Generalized   Min. cluster size:                  33
                      Estimating Equations   Max. cluster size:                  33
```

```
Family:                            Gaussian   Mean cluster size:                 33.0
Dependence structure:          Independence   Num. iterations:                      2
Date:                      Thu, 11 Apr 2019   Scale:                            0.000
Covariance type:                     robust   Time:                          16:20:30
====================================================================================
                        coef    std err          z      P>|z|      [0.025      0.975]
------------------------------------------------------------------------------------
Intercept             0.0042      0.000     12.084      0.000       0.004       0.005
diet[T.Fortasyn]   9.928e-05      0.000      0.222      0.824      -0.001       0.001
days_from_surgery  1.053e-05   8.32e-06      1.265      0.206   -5.78e-06    2.68e-05
==============================================================================
Skew:                          0.7832   Kurtosis:                          0.8481
Centered skew:                 0.5774   Centered kurtosis:                 0.8016
==============================================================================
END Overall GLM RESULTS
```
